# Supplementary material for: Absence of Light Exposure Increases Pathogenicity of Pseudomonas aeruginosa Pneumonia-Associated Clinical Isolates
Source: Biology (Basel). 2021 Aug 27;10(9):837. doi: 10.3390/biology10090837 (PMC8466069; doi:10.3390/biology10090837)
Supplement: Supplementary file 1 [file biology-10-00837-s001.zip › SupMaterial_revised.pdf]

Supplementary Material

Absence of light exposure increases pathogenicity of *Pseudomonas aeruginosa* pneumonia-associated clinical isolates

Cristina S. Mesquita[1], Artur Ribeiro[2], Andreia C. Gomes[1], Pedro Santos[1,*]

[1] CBMA – Centre of Molecular and Environmental Biology, Department of Biology, University of Minho, Campus of Gualtar, 4710-057 Braga, Portugal

[2] CEB - Centre of Biological Engineering, University of Minho, Campus of Gualtar, 4710-057, Braga, Portugal

[*] Correspondence: psantos@bio.uminho.pt

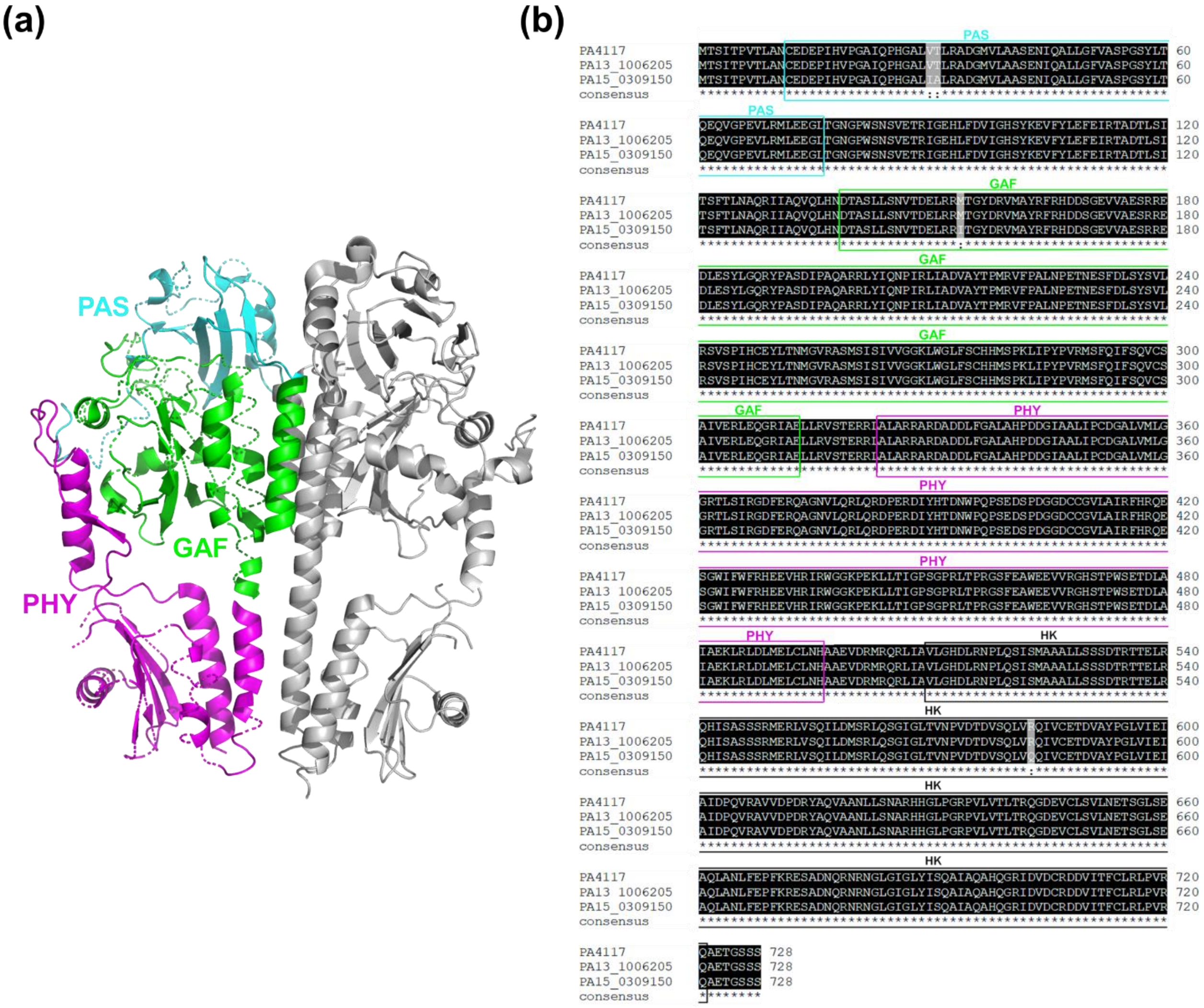

Figure S1. Bacteriophytochrome BphP: the photoreceptor identified in *P. aeruginosa* PAO1 found to be present in the clinical isolates HB13 and HB15 by in silico analysis. (**a**) Determined crystal structure of the photosensory core domain of the dimeric BphP (PDB: 3C2W) [1]. The Per-ARNT-Sim (PAS), cGMP phosphodiesterase/adenyl cyclase/FhlA (GAF) and phytochrome (PHY) domains of one monomer are highlighted in cyan, green, and magenta, respectively. A structural alignment between the C-terminal of this core domain and the N-terminal of the histidine kinase (HK) domain is proposed by the authors. The bacteriophytochrome BphP (PA13_1006205, PA15_0309150, PA4117) assembles with biliverdin, its chromophore, produced by the heme oxygenase BphO (PA13_1006200, PA15_0309145, PA4116) to generate a photosensing HK that is activated by light. (**b**) Sequence alignment with representation of the respective locus_tag. Boxes identify the residues corresponding to the four described domains: PAS, GAF, PHY and HK [1]. The "*" indicates positions which have a single, fully conserved residue (black); The ":" indicates the conservation between groups of strongly similar properties (grey). The numbering of each sequence represents the position of each residue in the original protein sequence. According to PROVEAN, none of the variations has a deleterious effect.

1. Yang, X.; Kuk, J.; Moffat, K. Crystal structure of *Pseudomonas aeruginosa* bacteriophytochrome: Photoconversion and signal transduction. *Proc. Natl. Acad. Sci.* **2008**, *105*, 14715–14720.

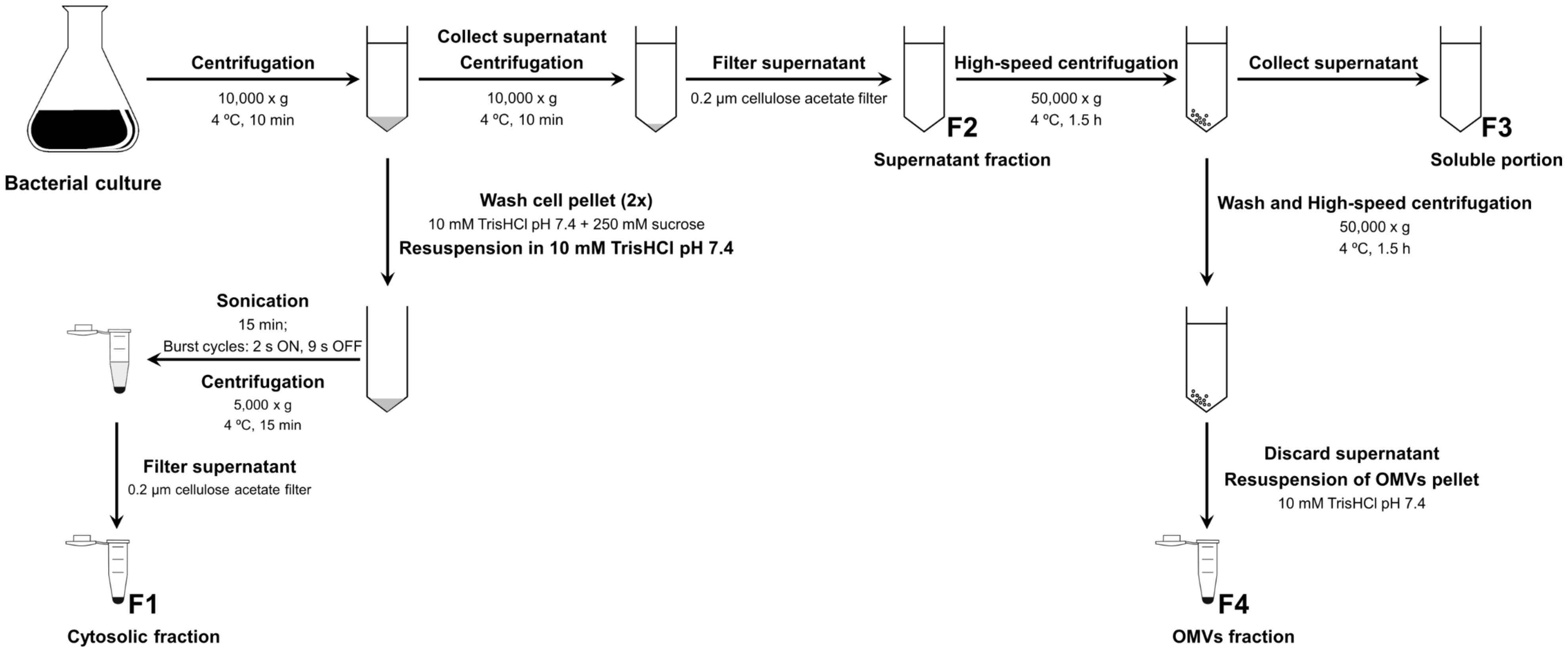

Figure S2. Procedure for collection of four different *P. aeruginosa* fractions. The reference strain PAO1 and the clinical isolates HB13 and HB15 were assayed. From each isolate, four fractions were collected, corresponding to the cytosolic fraction (F1), the whole supernatant (F2), the further fractioned soluble portion (F3) and the OMVs fraction (F4).

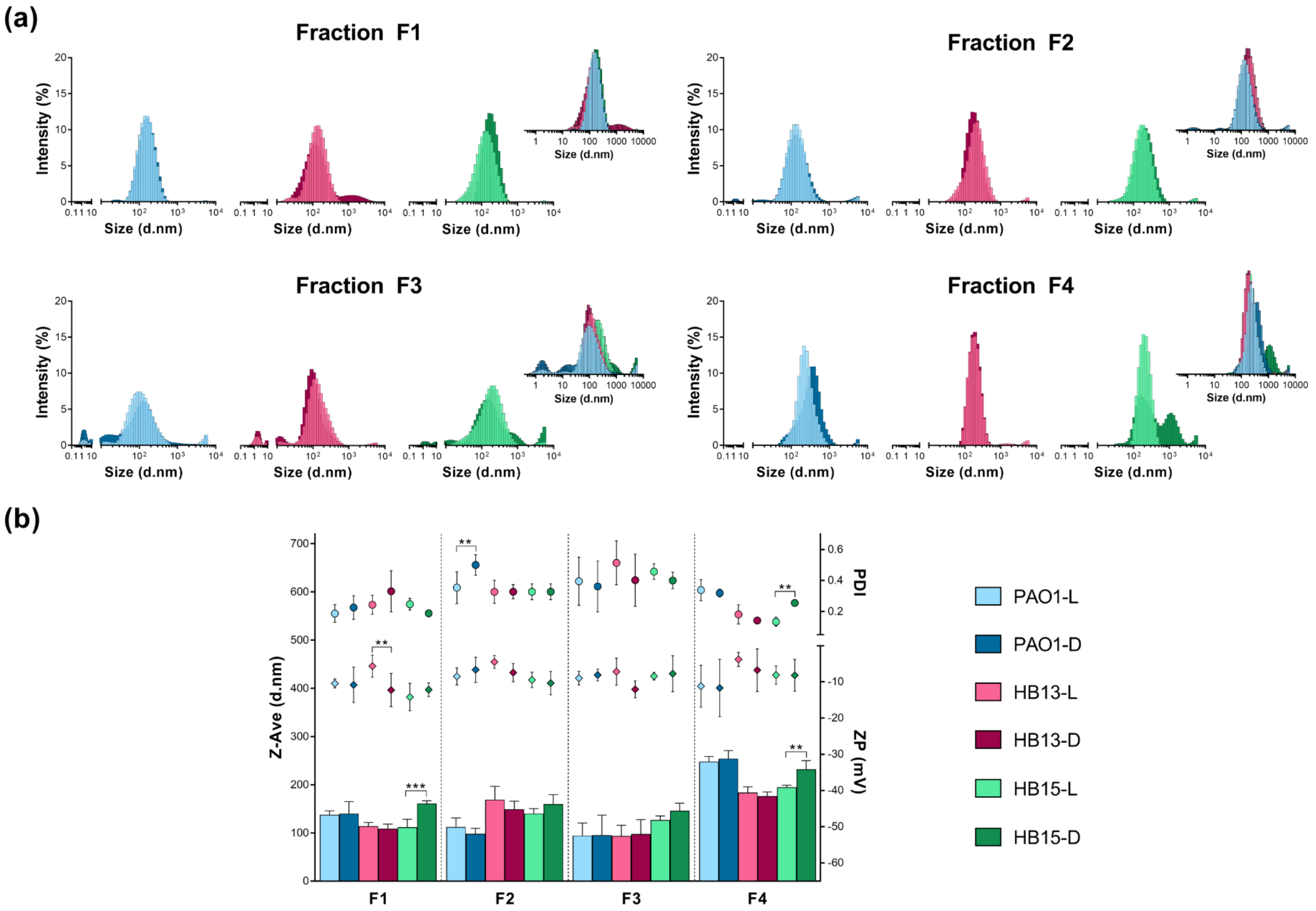

Figure S3. Characterization of the fractions of *P. aeruginosa* grown in constant exposure to full-spectrum light (L) and total absence of light (D) by DLS. (**a**) Particle size distribution by intensity organized by fraction. Combined view of the distribution is represented. (**b**) Analysis of physicochemical properties, particularly Z-ave, ZP and PDI. Standard deviation bars are represented for three independent experiments conducted in triplicate. Statistical comparisons performed by two-way ANOVA, followed by Tukey's post-hoc test for multiple comparisons. Significant differences comparing the two growth conditions are indicated as: ** $P < 0.01$, *** $P < 0.001$.

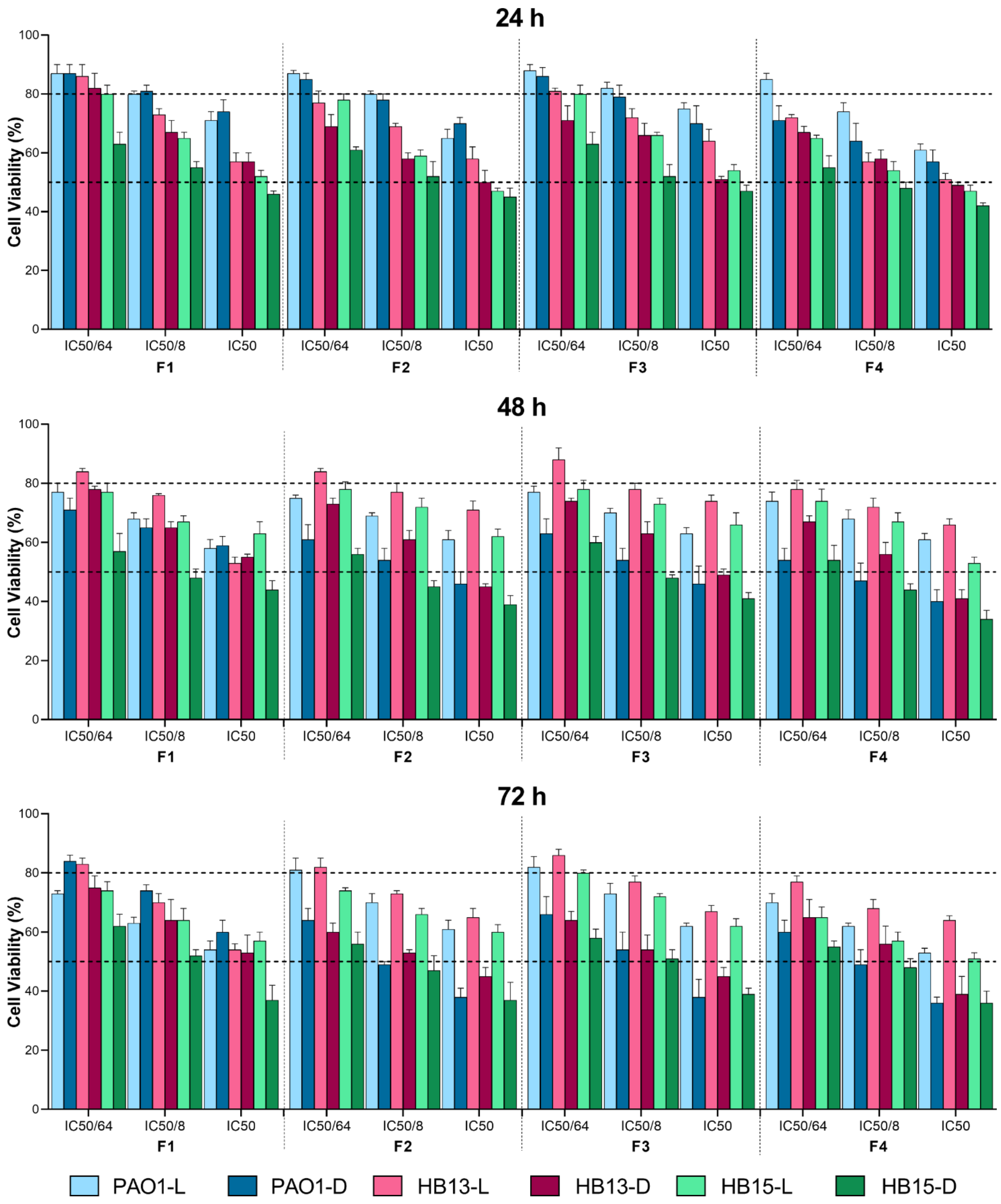

Figure S4. Effect on A549 cell viability (%) induced by fractions of *P. aeruginosa* grown in constant exposure to full-spectrum light (L) and total absence of light (D). Results of the MTT assay are depicted as the cell viability percentage of A549 cells exposed to a serial range of three concentrations (from a 64x dilution of the IC50 to the IC50) of each fraction. Horizontal lines highlight a cellular viability of 50% and 80%. Three MTT endpoints were assayed (24, 48 and 72 h of contact) and standard deviation bars are represented for three independent experiments.

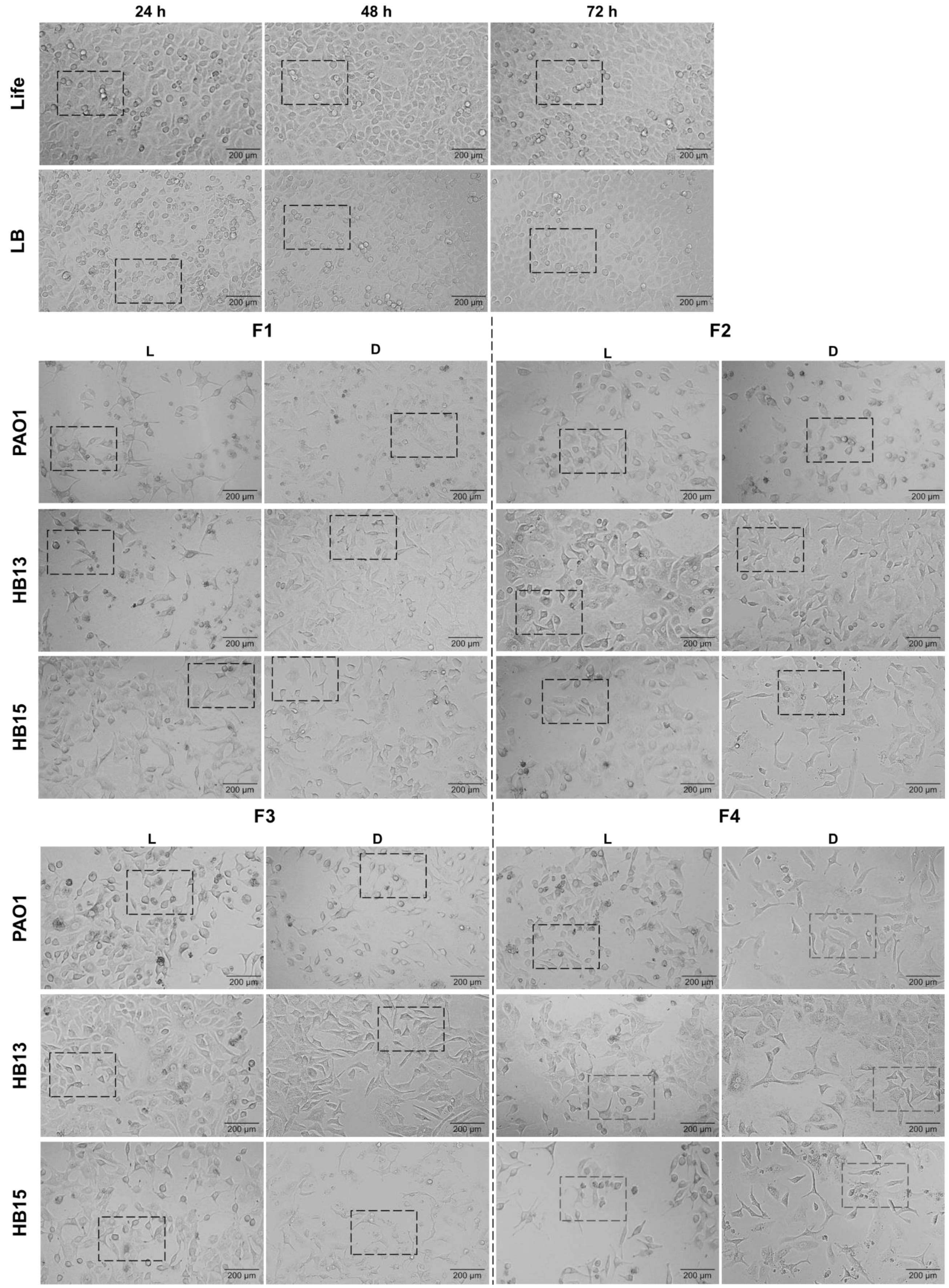

Figure S5. Contrast phase micrography of A549 cells exposed to a specific concentration (Table 1) at 48 h endpoint. Morphologic alterations induced in A549 cells by fractions of *P. aeruginosa* grown in constant exposure to full-spectrum light (L) and total absence of light (D). A representative Life control for the three MTT endpoints is presented. The scale bar is set for 200 µm. Section of the contrast phase micrography presented in Figure 2 is identified by a dashed rectangle.
