# Supplementary figures and images for: Absence of Light Exposure Increases Pathogenicity of Pseudomonas aeruginosa Pneumonia-Associated Clinical Isolates
Source: Biology (Basel). 2021 Aug 27;10(9):837. doi: 10.3390/biology10090837 (PMC8466069; doi:10.3390/biology10090837)

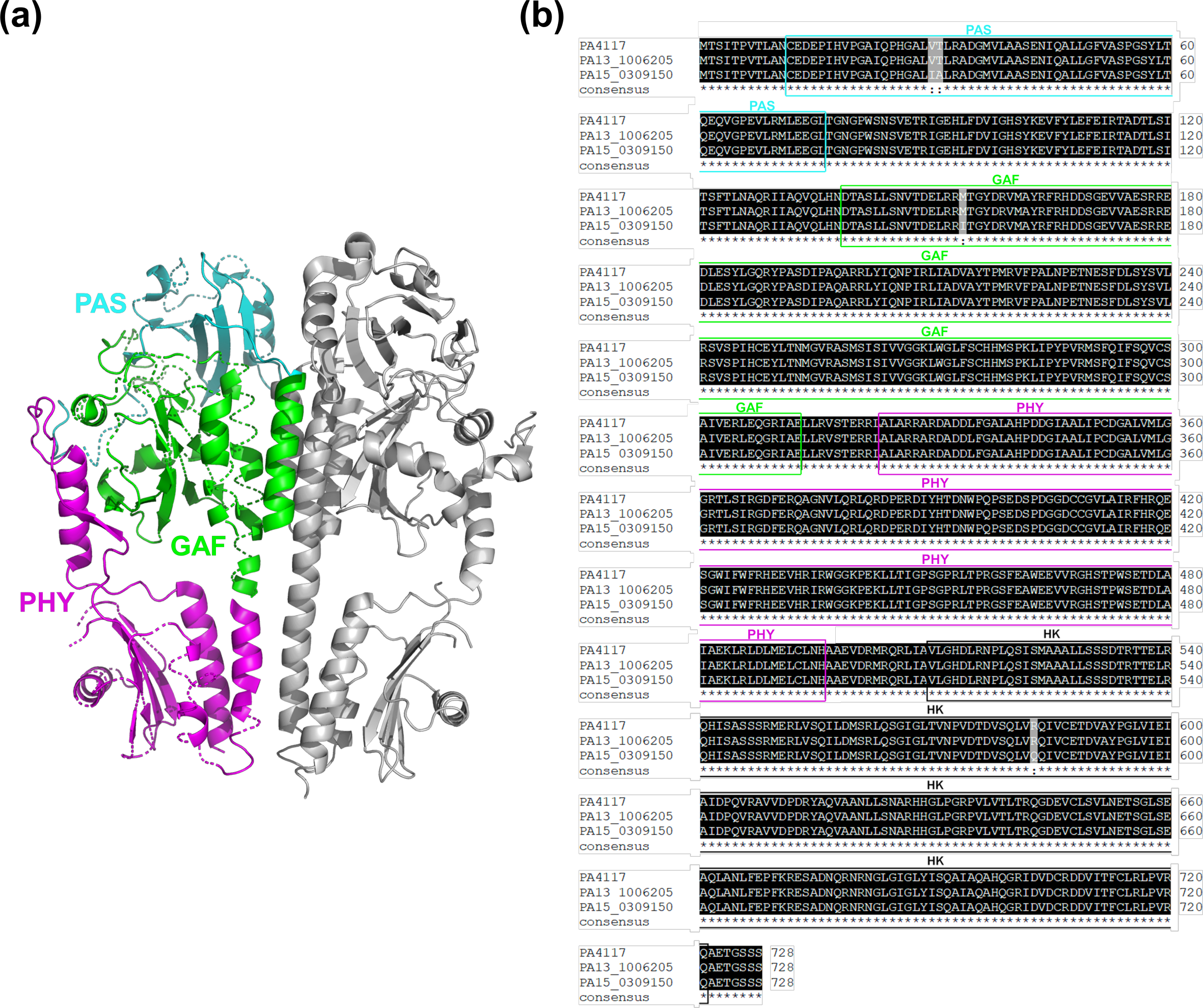

Supplement: Supplementary file 1 [file biology-10-00837-s001.zip › FigureS1.tif]

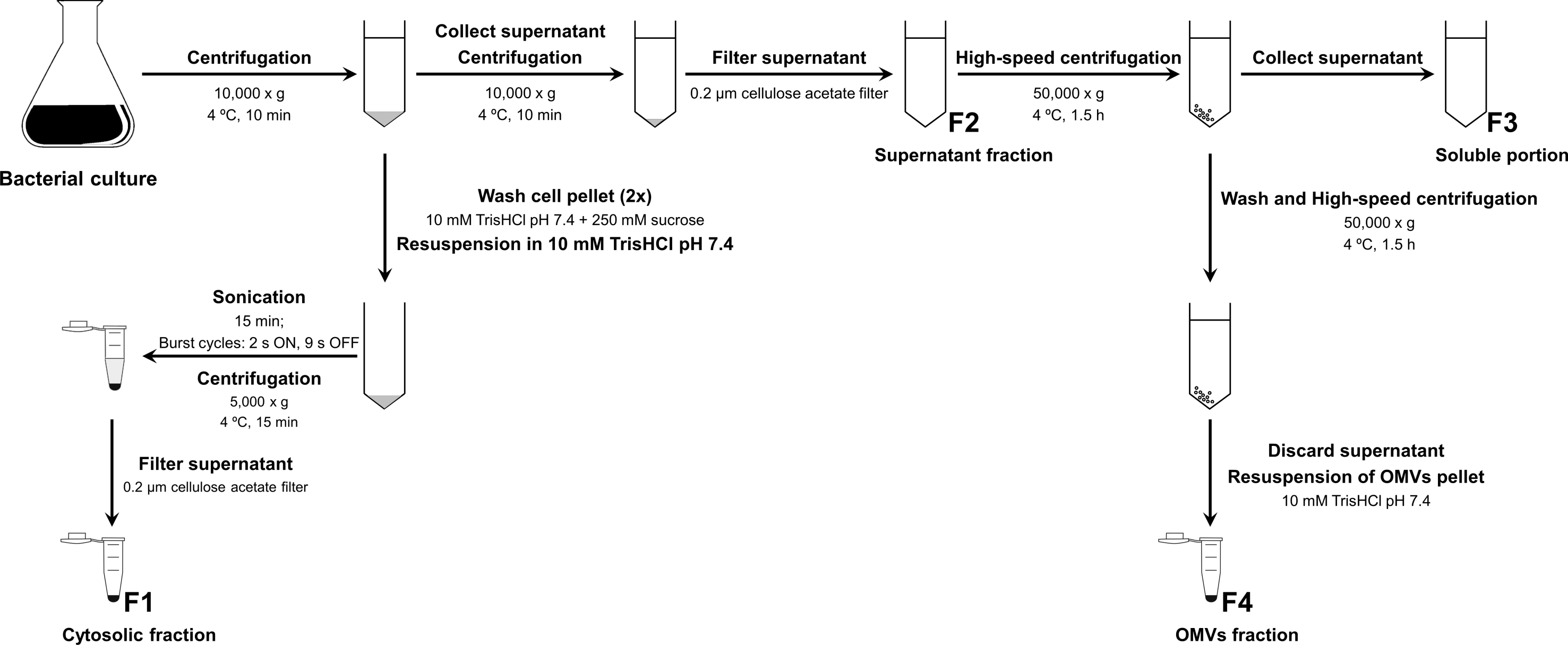

Supplement: Supplementary file 1 [file biology-10-00837-s001.zip › FigureS2.tif]

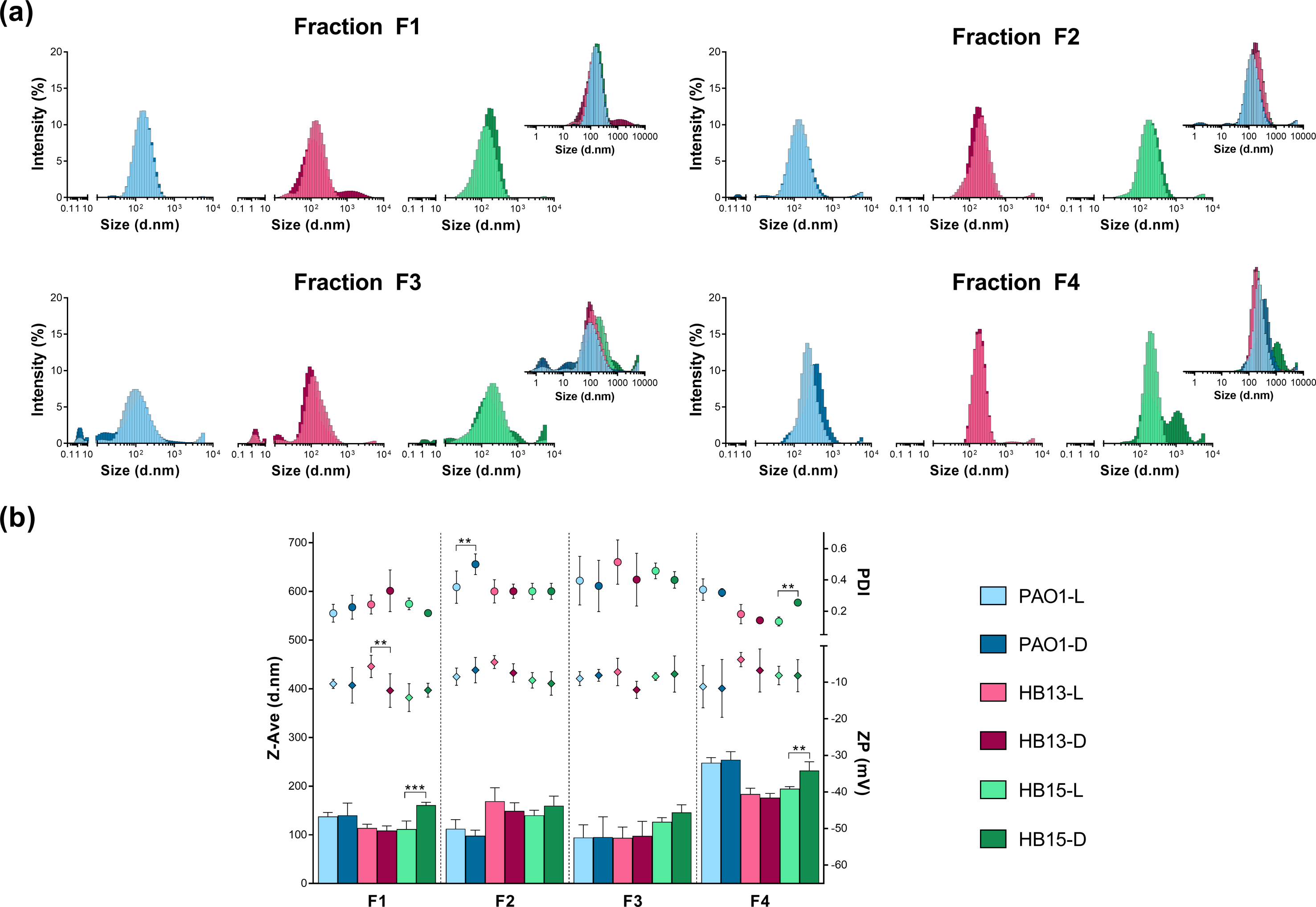

Supplement: Supplementary file 1 [file biology-10-00837-s001.zip › FigureS3.tif]

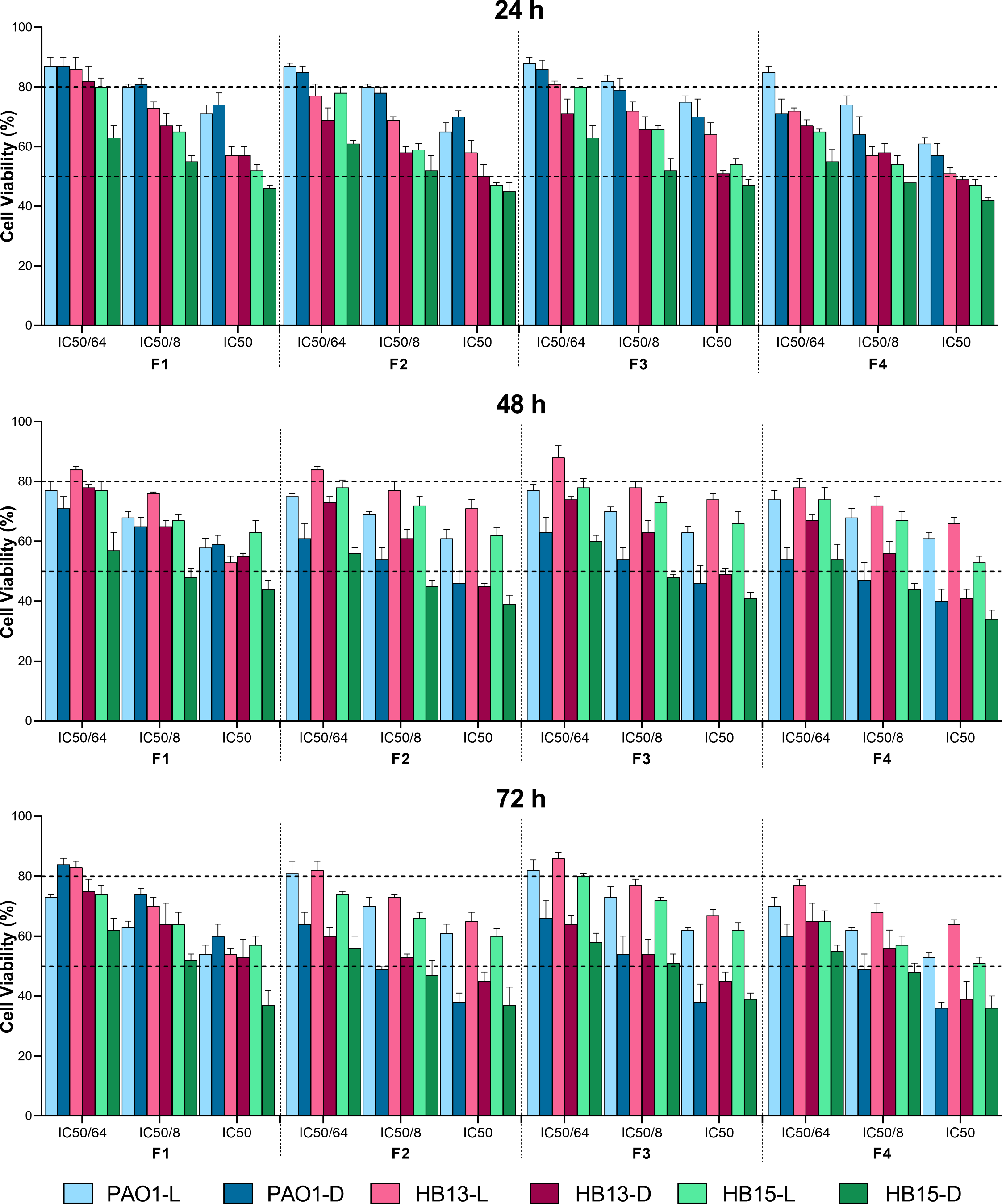

Supplement: Supplementary file 1 [file biology-10-00837-s001.zip › FigureS4.tif]

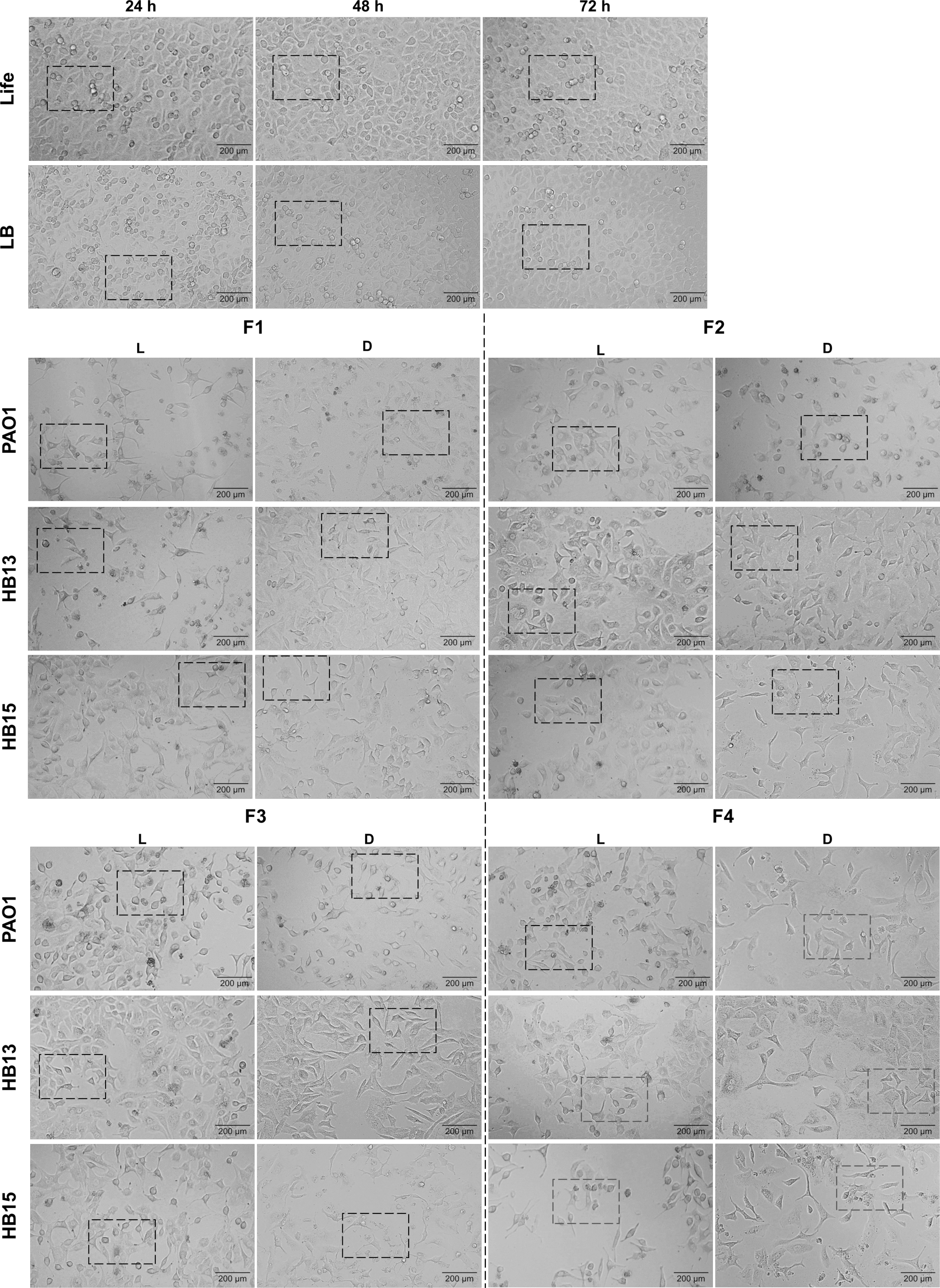

Supplement: Supplementary file 1 [file biology-10-00837-s001.zip › FigureS5.tif]
